# MiR-146a in Immunity and Disease

**DOI:** 10.4061/2011/437301

**Published:** 2011-04-07

**Authors:** Nicole Rusca, Silvia Monticelli

**Affiliations:** Institute for Research in Biomedicine, Via Vincenzo Vela 6, 6500 Bellinzona, Switzerland

## Abstract

MicroRNAs (miRNAs) are regulatory molecules able to influence all aspects of the biology of a cell. They have been associated with diseases such as cancer, viral infections, and autoimmune diseases, and in recent years, they also emerged as important regulators of immune responses. MiR-146a in particular is rapidly gaining importance as a modulator of differentiation and function of cells of the innate as well as adaptive immunity. Given its importance in regulating key cellular functions, it is not surprising that miR-146a expression was also found dysregulated in different types of tumors. In this paper, we summarize recent progress in understanding the role of miR-146a in innate and adaptive immune responses, as well as in disease.

## 1. Introduction

MicroRNAs (miRNAs) represent a pervasive feature of all cells, as they regulate large fractions of the cell's transcriptome. So far, 672 mouse miRNAs and 1048 human miRNAs have been described in the miRBase database (http://www.mirbase.org/, release Sept. 2010) with each miRNA potentially regulating the expression of hundreds of target genes, highlighting the extent of this form of regulation [1]. Whereas some miRNAs are widely expressed, others exhibit only limited developmental stage-, tissue-, or cell type-specific patterns [2]. Similar to any other mammalian cell type, cells of the immune system rely on miRNAs to regulate lineage commitment, proliferation, migration, and differentiation. In most cases, these activities are orchestrated by both ubiquitously expressed and cell type-specific miRNA species [3–7]. The importance of miRNAs in regulating differentiation and function of immune cells is underlined by the phenotypical perturbations that occur when miRNA expression is altered. Given the emerging roles of miRNAs in modulating immune responses, it is likely that any dysregulation of miRNA expression may contribute to the pathogenesis of autoimmune diseases, chronic inflammation, and malignancies. Indeed, several human diseases have now been associated with dysregulated miRNA expression, and miRNAs have been shown to function both as oncogenes and tumor suppressor genes [8, 9]. MiR-146a has been recently shown to be an important modulator of differentiation and function of cells of innate as well as adaptive immunity. Here, we summarize recent progress in understanding the role of miR-146a in immune responses and in disease (see also Table 1).

## 2. What Are MicroRNAs?

MiRNAs are small (20–25 nucleotides), noncoding RNA molecules involved in posttranscriptional gene regulation. They derive from primary transcripts (pri-miRNA) that are processed into hairpin precursors (pre-miRNAs) within the nucleus of the cell by the Microprocessor complex, which includes the RNAseIII enzyme Drosha. Pre-miRNAs are translocated into the cytoplasm and processed by Dicer into their mature form (for a recent review see [25]). An exception to this rule is represented by the less abundant “mirtrons”, that bypass Drosha and are processed only by Dicer [26]. Mature miRNAs loaded onto the RNA-induced silencing complex (RISC) recognize sites located mostly in the 3′ untranslated region (3′ UTR) of target mRNAs through canonical base-pairing between the seed sequence of the miRNA (nucleotides 2–8 at its 5′ end) and its complementary sequence in the target mRNA. This leads to a block in translation with or without destabilization and degradation of the targeted mRNA. MiRNAs modulate a broad range of gene expression patterns during development and homeostasis, as well as in pathogenesis of disease. Most miRNAs are transcribed by RNA polymerase II, and their upstream regulatory regions contain canonical core promoters and enhancers, regulated by transcription factors. The importance of miRNA biogenesis as a whole in the immune system is clearly highlighted by the fact that conditional ablation of Dicer or other miRNA processing factors resulted in a profound block of both B- and T-cell development [27–30]. Moreover, deletion or overexpression of certain individual miRNAs also led to a severe impairment of the development and/or function of cells of the immune system [31–38]. Some miRNAs, like miR-146, are expressed as a family that shares the same seed sequence, but is encoded by different loci in the genome. Indeed, the miR-146a gene is located on mouse chromosome 11, while miR-146b is located on chromosome 19 (chromosomes 5 and 10, resp., in human). The mature sequences for miR-146a and miR-146b differ by only two nucleotides. Nevertheless, since they share the same seed sequence they should in principle recognize the same targets [39]. While in some cell types (like monocytes) these miRNAs appear to have similar functions [12], in other cases, like in regulatory T (Treg) cells [10], only miR-146a, but not miR-146b, was shown to be highly expressed. While it is still unclear if these two miRNAs have redundant and/or separate functions, the majority of the published work focuses mostly on miR-146a, which is therefore the miRNA we will be mainly referring to in this paper.

## 3. MiR-146a in Adaptive Immune Responses

Adaptive immune responses are vital for the efficient eradication of infectious agents, although dysregulated responses might also lead to autoimmune and chronic inflammatory diseases. The development and propagation of an adaptive immune response specific for an invading pathogen is a highly orchestrated process that involves the activation and proliferation of immune cells and their subsequent migration to sites of inflammation. The first indication that miRNAs were involved in regulating differentiation of cells in the immune system came from a study from Chen and colleagues that identified miR-181 as a miRNA specifically expressed in hematopoietic cells [31]. Its ectopic expression in hematopoietic progenitors led to an increased fraction of B-lineage cells in both *in vitro* differentiation assays and mouse models. Following this pioneering work, many studies identified miRNAs as crucial components of the molecular circuitry that controls differentiation and functions of cells of the immune system.

Upon encounter with the antigen, naïve CD4+ T-cells give rise to T-cell subsets (Th1, Th2, Th17, Tregs, T follicular helper and probably others) with functions that are tailored to their respective roles in host defence [40]. Initial expression profiling studies identified miRNAs specifically expressed in different T-cell subsets and stages of differentiation [5–7]. T-cell-specific deletions of Dicer revealed a requirement for the miRNA pathway in the development of T cells [27, 29], as well as for differentiation of effector T-cell subsets. Indeed, T cells lacking Dicer showed increased differentiation to the Th1 subset with a correspondingly reduced polarization to Th2 [29]. Adding to the complexity of gene regulatory networks, proliferating T cells express genes with shorter 3′ UTRs than those expressed in resting T cells, making these mRNAs less susceptible to regulation by miRNAs due to the loss of miRNA binding sites [41]. Finally, individual miRNAs were also shown to play important roles in T-cell differentiation and function. For example, miR-181a, which is upregulated during T-cell development, was shown to enhance T-cell receptor (TCR) signalling strength by directly targeting a number of protein phosphatases [32], while mice lacking miR-155 showed an altered Th1/Th2 polarization with a bias towards Th2, indicating that miR-155 promotes differentiation towards Th1 cells [35]. 

As for the role of miR-146a in T cells, by analyzing the expression of miRNAs in highly purified subsets of cells of the immune system, we showed that miR-146a is one of the very few miRNAs differentially expressed between Th1 and Th2 cells in the mouse, suggesting that it might be involved in fate determination of these cells [5]. Recent work performed in miR-146a-deficient mice showed an increase in the percentage of INF*γ*-producing T-cell subset in the absence of miR-146a [10]. In human T cells, miR-146a is expressed at low levels in naïve T lymphocytes while it is abundantly expressed in memory T cells and it is induced upon TCR stimulation, consistent with its expression being dependent on NF-*κ*B induction [12, 13]. Indeed, NF-*κ*B and c-ETS-binding sites were shown to be required for the induction of miR-146a transcription in human T cells, and such induction potentially modulated cell death in these cells by targeting FADD and by impairing both AP-1 activity and IL-2 production [13]. Treg cells constitute a specialized T-cell subset able to maintain immune homeostasis by limiting the inflammatory responses, and their suppressive function is indispensable for immune homeostasis and survival of higher organisms. Recently, Lu and colleagues reported that miR-146a is among the miRNAs prevalently expressed in Treg cells and showed that it is critical for Treg functions. Indeed, deficiency of miR-146a resulted in increased numbers but impaired function of Treg cells and as a consequence, breakdown of immunological tolerance with massive lymphocyte activation, and tissue infiltration in several organs [10]. The immune-mediated lesions induced by the lack of miR-146a in Tregs were dependent on INF*γ* and Stat1.

## 4. MiR-146 in Innate Immunity and Nonimmune Systems

**Figure 1:**
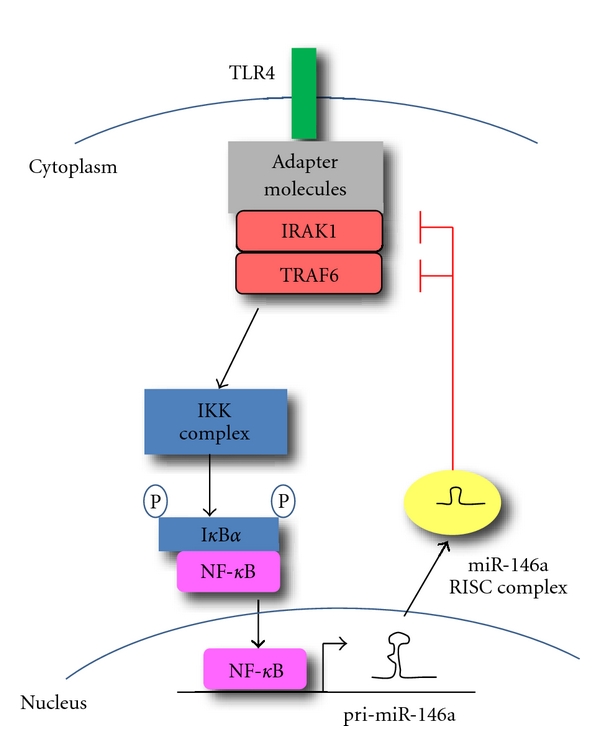
MiR-146a negatively regulates signal transduction pathways leading to NF-*κ*B activation. Upon activation of a cell surface receptor such as TLR4, a molecular cascade including TRAF6 and IRAK1 leads to I*κ*B*α* phosphorylation and degradation and to NF-*κ*B activation and nuclear translocation [12, 42]. NF-*κ*B activation induces transcription of many genes, including pri-miR-146a. Once translocated to the cytoplasm and loaded onto the RISC complex, mature miR-146a contributes to attenuate receptor signaling through the downmodulation of IRAK1 and TRAF6.

Cells of the innate immune system, such as granulocytes, natural killer (NK) cells, monocytes, and macrophages, provide an important first line of defense for the organism against invading pathogens. MiRNAs have been implicated in both the development and functions of innate immune cells. For example, the macrophage inflammatory response to infection involves the upregulation of several miRNAs, such as miR-155, miR-146, miR-147, miR-21, and miR-9 [12, 43–46]. Several studies linked miR-146a expression to NF-*κ*B signaling within the innate immune system (Figure 1) and were initiated by a study showing that miR-146a is quickly induced upon activation of human monocytes [12]. In this study, miR-146a was found to be inducible upon stimulation with LPS in a NF-*κ*B-dependent manner, and to target the TNF receptor-associated factor 6 (TRAF6) and IL-1 receptor-associated kinase 1 (IRAK1) genes. These genes encode two key adapter molecules downstream of cytokine and Toll-like receptors (TLR), pointing towards a role for miR-146a in controlling signaling from these receptors through a negative feedback regulatory loop involving downregulation of TRAF6 and IRAK1 [12]. It was also suggested that miR-146a contributes to the establishment of endotoxin tolerance in monocytes and to the regulation of TNF*α* production [14]. In this context, miR-146a would therefore act as a tuning mechanism to prevent an overstimulated inflammatory state. In human Langerhans cells (LCs), miR-146a was found to be constitutively expressed at high levels, as compared to interstitial dendritic cells (intDCs) [15]. In these cells, high miR-146a expression was induced by the transcription factor PU.1 in response to TGF-*β*1, a key signal for epidermal LC differentiation, and while it did not influence myelopoiesis or DC subset differentiation, the authors suggested that constitutively high miR-146a expression may represent a novel mechanism to desensitize LCs to inappropriate TLR signaling at epithelial surfaces through decreased NF-*κ*B signal strength downstream of the receptor [15]. 

Somewhat differently from the studies mentioned above, a study performed in human lung alveolar epithelial cells showed a rapid, time- and concentration-dependent increase in miR-146a upon stimulation with IL-1*β* [16]. Such increased miR-146a expression negatively regulated the release of the proinflammatory chemokines IL-8 and RANTES in a way that did not seem to involve IRAK1 and TRAF6, highlighting how the role of miRNAs can be exquisitely cell type-specific. MiR-146a was also shown to have an important role in normal epithelial functions: specifically, miR-146a-mediated downregulation of IRAK1 was sufficient to induce innate immune tolerance and provide protection from bacteria-induced epithelial damage in neonates [11].

A molecular cascade involving miR-146a, the miR-146a negative regulator PLZF, and the miR-146a target CXCR4 was also shown to be active during megakaryopoiesis [17]. This regulatory pathway involved enhanced expression of PLZF, which in turn inhibited miR-146a transcription. The resulting downmodulation of miR-146a caused an increase in CXCR4 expression, which is necessary for megakaryocytes differentiation and maturation. As a result, miR-146a overexpression, as well as PLZF or CXCR4 silencing, impaired megakaryocytic proliferation, differentiation, and maturation, as well as colony formation [17].

## 5. MiR-146a and Viruses

Various layers of negative regulators are used by immune cells to avoid uncontrolled immune responses when facing viral invasion, and such regulatory mechanisms can also be used by viruses in order to escape immune surveillance. A role for miR-146a was discovered in the regulation of vesicular stomatitis virus (VSV) infection [23]. In macrophages, VSV infection upregulated miR-146a expression in a RIG-I/NF-*κ*B-dependent manner. Elevated miR-146a expression led to negative regulation of the production of VSV-triggered type I IFN through downregulation of TRAF6, IRAK1, and IRAK2, thus promoting VSV replication in macrophages [23]. The authors proposed a model in which VSV infection is first sensed by RIG-I, which in turn initiates type I IFN production against VSV infection. At the same time, VSV infection upregulates miR-146a expression, which inhibits innate antiviral immune response by impairing RIG-I signaling. 

The Epstein-Barr virus (EBV) infects over 90% of the human population worldwide. EBV infection can result in a number of malignancies, including Burkitt's and Hodgkin's lymphomas. LMP1 (latent membrane protein 1) is the major oncoprotein of EBV, able to activate transcription factors such as NF-*κ*B and AP-1, and thus to manipulate host cellular processes that regulate cell proliferation, migration, and apoptosis. Through its ability to activate transcription factors, LMP1 also induces expression of cellular miRNAs, most notably miR-146a, which therefore could contribute to cellular immortalization and tumorigenesis in the context of EBV infection [24].

## 6. MiR-146 and Cancer

Cancer is the result of a complex multistep process that involves the accumulation of sequential alterations in several genes, including those encoding miRNAs [8, 9]. Since miRNAs participate in keeping the balance of gene regulatory networks that determine the fate of a cell, their dysregulation potentially weakens this balance, thereby contributing to oncogenesis and cancer progression. Indeed, miRNA profiling has uncovered significantly altered miRNA expression in many types of cancer [8]. 

Initial evidences on the possible involvement of miR-146a in cancer came from a study showing that miR-146a was upregulated in papillary thyroid carcinoma (PTC) samples compared with unaffected thyroid tissue. Interestingly, a set of five miRNAs, including miR-221, miR-222, and miR-146, was sufficient to distinguish unequivocally between PTC and normal thyroid [47]. Similarly to the observations performed in immunologic settings, overexpression of miR-146a/b in the highly metastatic human breast cancer cell line MDA-MB-231 significantly downregulated expression of IRAK1 and TRAF6, negatively regulating NF-*κ*B activity [18]. Functionally, this resulted in markedly impaired invasion and migration capacity relative to control cells. These findings implicated miR-146 not only as a negative regulator of constitutive NF-*κ*B activity in breast cancer cells, but also suggested that modulating miR-146 levels might have therapeutic potential to suppress breast cancer metastases. Along the same line, miR-146a was among the miRNAs found upregulated in cervical cancer tissues compared to normal cervix [19]. When introduced into cell lines, miR-146a promoted cell proliferation. Although the molecular mechanism underlying such increased proliferation remains to be investigated, these observations potentially implicate miR-146a in cervical carcinogenesis. In another type of cancer, the hormone-refractory prostate carcinoma (HRPC), miR-146a levels were diminished compared to androgen-sensitive noncancerous epithelium [20]. In this context, miR-146a acted as a tumor suppressor, reducing levels of its target ROCK1, one of the key kinases involved in HRPC transformation. Accordingly, forced miR-146a expression reduced ROCK1 protein levels, cell proliferation, invasion, and metastasis to human bone marrow endothelial cell monolayers. Similarly, miR-146a was lower in pancreatic cancer cells compared with normal human pancreatic cells [21]. Re-expression of miR146a inhibited the invasive capacity of pancreatic cancer cells with downregulation of EGFR (epidermal growth factor receptor) and IRAK-1. Finally, a recent study showed that the treatment of bone marrow-derived mesenchymal stem cells (MSCs) with diazoxide (DZ) markedly increased the expression of miR-146a and promoted cell survival. Moreover the downregulation of miR-146a expression by antisense inhibitors eliminated the DZ-induced cytoprotective effects. This result suggested a critical role of miR-146a in MSC survival [48].

## 7. Polymorphisms and Posttranscriptional Modifications

Polymorphisms affecting miRNA expression, maturation, or mRNA recognition may also become important determinants for increased tumor risk. Indeed, a genetic variant in the 3′ UTR of the KIT oncogene was recently described, that resulted in a mismatch in the seed region of miR-221 and correlated with increased risk of melanoma [49]. As for miR-146a, a single nucleotide polymorphism (rs2910164; G/C) was found on the passenger strand of pre-miR-146a [22]. The rarer C allele decreased nuclear pri-miR-146a processing, reducing levels of pre-miR-146a and mature miR-146a and unblocking expression of its target genes, including TRAF6 and IRAK1. In an association study of PTC patients, the germ-line GC heterozygous state was associated with an increased risk of acquiring PTC, while both homozygous states (GG and CC) were protective. Importantly, this polymorphism was also found to undergo somatic mutation in PTC tumor tissue [22], underlying the need for more studies on polymorphisms, both on the miRNAs themselves as well as on the binding sites in their targets as, based on the studies mentioned above, they can clearly contribute to cancer progression. 

Along the same line, point mutations in either a miRNA or its targets may dramatically alter miRNA expression and/or functionality, respectively. Even in normal, nondiseased conditions, specific adenosine residues of certain miRNA precursors can be edited by adenosine deaminase acting on RNA enzymes (ADAR) [50]. The resulting A → I conversions replace A-U Watson-Crick pairs with I : U wobble pairs in the double-stranded RNA, altering miRNA processing. For example, editing of pri-miR-142, expressed in hematopoietic tissues, resulted in suppression of its processing by Drosha and consequently in its degradation [50]. Interestingly, interferons induce the upregulation of ADAR1 [51], thus raising the possibility that mutations introduced by ADAR in the pri-miRNAs might lead to alteration of miRNA expression and/or target recognition during inflammation [52].

## 8. miR-146: A Role as Biomarker?

A biomarker is a measurable indicator of a biological state, either normal or pathological, with or without pharmacological treatments [53]. Ideal biomarkers should be detectable with high sensitivity and specificity, should have high predictive power, and should be accessible in a noninvasive manner. The data accumulated on miRNA expression in tumors demonstrate that miRNAs are indeed promising candidates to distinguish between different tumors and different subtypes of tumors, as well as to predict their clinical behavior [53]. Large miRNA expression studies have supported the role of miRNAs as either prognostic and/or diagnostic markers in various types of cancer (for a detailed review see [8]). These studies generally showed that the miRNA profiles reflected the developmental lineage and the differentiation status of tumors, and that such miRNA signatures enabled the tumor samples to be grouped on the basis of their tissue of origin. Such profiling studies will become a useful tool to identify miRNA signatures that are associated with a particular diagnosis or probable outcome of a disease. 

As mentioned above, a good biomarker should also be easily accessible; the ability to detect clinically relevant miRNAs in the plasma or serum of patients raises therefore a lot of interest, particularly since serum miRNAs appear to exist in a stable and protected form, possibly within exosome-like particles [53, 54]. Moreover, the levels of miRNAs in serum were shown to be stable, reproducible, and consistent among individuals of the same species [55]. While more studies need to be performed on the role that miR-146a specifically might have as a biomarker, there is little doubt that miRNA detection in serum or PBMCs could provide a convenient and noninvasive measure for diagnosis and monitoring of many different types of disease.

## 9. Concluding Remarks

The discovery of miRNAs has revealed a new layer of regulation of gene expression with a profound impact on many biological systems. Studies in recent years have shown that miRNAs have a unique expression profile in cells of the innate and adaptive immune system and have crucial roles in the regulation of both cell development and function. Moreover, it is becoming widely accepted that miRNAs can function both as oncogenes or tumor suppressors in an expanding number of tumors and cell types. There is now increasing evidence to suggest that miR-146a is involved in the regulation of the adaptive as well as innate immune response, and that miR-146a can be an important player in regulating tumor progression. However, more work remains to be done to fully understand its role and mechanism of action in normal and pathologic conditions, so that expression of this miRNA can potentially be exploited as a new point of entry for therapy. With the identification of a vast number of miRNAs each carrying a long list of putative targets, the challenge is now to understand the details of their biological functions.

## Figures and Tables

**Table 1:** Summary of the described roles for miR-146a in immune responses and disease, with indicated references and known targets.

Ref.	Expression/Function	Targets
	*Mouse*	
[5]	Differential expression in Th1/Th2 cells	
[10]	Impaired Treg function in mice lacking miR-146a	STAT1
[11]	Provided protection from bacteria induced epithelial damage in neonates	IRAK1

	*Human*	
[12]	Attenuated TLR4 signaling in monocytes	IRAK1, TRAF6
[13]	Regulated activation induced cell death and IL-2 expression in Jurkat T cells	FADD
[14]	Contributed to the establishment of endotoxin tolerance in monocytes	IRAK1, TRAF6
[15]	Desensitized Langerhans cells to inappropriate TLR signaling	
[16]	Negatively regulated inflammatory response in lung epithelial cells	
[17]	Controlled megakaryopoiesis	CXCR4
[18]	Reduced migration and invasion capacity of breast cancer cells	IRAK1, TRAF6
[19]	Promoted cell proliferation in cervical cancer	
[20]	Tumor suppressor in hormone-refractory prostate cancer	ROCK1
[21]	Suppressed invasion of pancreatic cancer cells	EGFR, IRAK1
[22]	SNP in pre-miR-146a predisposes to papillary thyroid carcinoma	

	*Viruses*	
[23]	Promoted VSV replication in macrophages	IRAK1, IRAK2, TRAF6
[24]	EBV-encoded LMP1 induced cellular miR-146a expression	
